# MAP4K4 and cancer: ready for the main stage?

**DOI:** 10.3389/fonc.2023.1162835

**Published:** 2023-05-08

**Authors:** Jaime González-Montero, Carlos I. Rojas, Mauricio Burotto

**Affiliations:** Bradford Hill Clinical Research Center, Santiago, Chile

**Keywords:** MAP4K4, MAP kinases, cytoskeleton, cancer, RNA interference

## Abstract

MAP4K4 is a serine/threonine kinase that belongs to the MAP kinase family and plays a critical role in embryogenesis and cellular migration. It contains approximately 1,200 amino acids and has a molecular mass of 140 kDa. MAP4K4 is expressed in most tissues where it has been examined and its knockout is embryonic lethal due to impaired somite development. Alterations in MAP4K4 function have a central role in the development of many metabolic diseases such as atherosclerosis and type 2 diabetes, but have recently been implicated in the initiation and progression of cancer. For example, it has been shown that MAP4K4 can stimulate the proliferation and invasion of tumor cells by activating pro-proliferative pathways (such as the c-Jun N-terminal kinase [JNK] and mixed-lineage protein kinase 3 [MLK3] pathways), attenuate anti-tumor cytotoxic immune responses, and stimulate cell invasion and migration by altering cytoskeleton and actin function. Recent *in vitro* experiments using RNA interference-based knockdown (miR) techniques have shown that inhibition of MAP4K4 function reduces tumor proliferation, migration, and invasion, and may represent a promising therapeutic approach in many types of cancer such as pancreatic cancer, glioblastoma, and medulloblastoma, among others. Over the last few years, specific MAP4K4 inhibitors such as GNE-495 have been developed but have not yet been tested in cancer patients. However, these novel agents may be useful for cancer treatment in the future.

## Introduction

MAP4K4 is a serine/threonine kinase that belongs to the mammalian family of Ste20 protein kinases. The members of this family can be divided into two groups based on the locations of their catalytic domains (1) and can be divided into eight subfamilies based on the structures of their non-catalytic regions (2). MAP4K4 is one of the four members of the germinal center-like kinase IV family (3). MAP4K4 kinase contains approximately 1,200 amino acids and has a molecular mass of approximately 140 kDa (4). The *MAP4K4* gene is located at 2q11.2 (1). MAP4K4 has multiple physiological functions and is expressed in all cell types where it has been examined, but its expression is highest in testicular tissue and cells of the nervous system (5). MAP4K4 knockout is lethal due to altered embryonic development and impaired cell migration (6). Furthermore, MAP4K4 has critical roles in the regulation of cell adhesion (7) and inflammation (8), and has been implicated in the development of metabolic diseases such as type 2 diabetes (9) and atherosclerosis (10).

Recent research has shown that MAP4K4 plays a role in cancer development and its inhibition may be a novel treatment strategy for several types of cancer. In this mini-review, we will discuss the available evidence underlying the role of MAP4K4 in cancer and the experimental data supporting its inhibition as a new therapeutic strategy.

## Role of MAP4K4 in cancer development

In recent years, several studies have reported that MAP4K4 plays a role in the initiation and progression of cancer. MAP4K4 is overexpressed in multiple tumor types such as pancreatic cancer (11), colorectal cancer (12), ovarian epithelial cancer (13), lung cancer (14), gastric cancer (15), and hepatocellular carcinoma (16). MAP4K4 contributes to cancer development in many ways but primarily acts through three main axes: activation of cell proliferation pathways, alteration of cytoskeleton function, and impairment of anti-tumor immune responses.

MAP4K4 can activate downstream pathways that promote tumor cell proliferation. A recent investigation demonstrated that MAP4K4-mediated phosphorylation and activation of mixed-lineage protein kinase 3 (MLK3) promoted pancreatic tumor proliferation, migration, and colony formation (17). Similarly, in an *in vitro* model of ovarian cancer, knockdown of MAP4K4 inhibited the migration of various cell types. The migration-promoting effect of MAP4K4 was mediated by c-Jun N-terminal kinase (JNK) and dependent on AP-1 activation (18). The ability of MAP4K4 to phosphorylate JNK was also demonstrated in an *in vitro* model of colorectal cancer (19). Additionally, knockdown of MAP4K4 inhibited the proliferation, growth, and migration of adenocarcinoma cells *in vitro*. Furthermore, In EGFR-mutated and erlotinib-treated lung adenocarcinoma cell lines, downregulation of MAP4K4 prevented ERK reactivation, suggesting that MAP4K4 is critical for maintaining tumor growth (20).

MAP4K4 can also alter cytoskeletal function. c-Met is a tyrosine kinase receptor aberrantly expressed in some types of medulloblastoma. It has been shown that MAP4K4 can control c-Met endocytosis and integrin-B1 activation, which are associated with invasive phenotypes in this tumor type (21). Similarly, Kumar et al. demonstrated that MAP4K4 mediated c-Met-induced invasive cell phenotypes by controlling actin dynamics in the cytoskeleton (22). Finally, experiments with CRISPR-Cas9 in an *in vitro* model of glioblastoma multiforme demonstrated that MAP4K4 was involved in cell motility and tumor invasion (23).

MAP4K4 can also impair anti-tumor immune responses. For example, genetic deletion of MAP4K4 has been shown to increase the expression of lymphocyte-associated antigen 1 (LFA1) on CD8^+^ T lymphocytes and improves their adherence to antigen-presenting cells (24). MAP4K4 deletion also increases CD8^+^ T lymphocyte activity, cytokine production, and cytotoxic activity. The interaction between MAP4K4 and LFA1 is mediated by ERM proteins (ezrin, radixin, and moesin) and could represent a new therapeutic opportunity in tumors with primary or acquired immunotherapy resistance. Further studies should address the role of MAP4K4 inhibition in the anti-tumor effects of immunotherapy. **Table 1** shows a summary about these 3 main ways by which MAP4K4 can stimulate tumor growth.

**Table 1:** Summary of the main ways in which MAP4K4 could stimulate tumor cell growth.

Affected function	Cancer type	Affected pathway	Reference
Activation of downstream pathways that promote tumor cell proliferation	Pancreatic cancer	Activation of MLK3	17
	Ovarian cancer	JNK and AP-1 activation	18
	Colorectal cancer	JNK	19
	Non-small cell lung cancer	ERK	20
Alteration of cytoskeletal function	Medulloblastoma	c-Met endocytosis and integrin-B1 activation	21
	Glioblastoma multiforme	Cell motility and tumor invasion	23
Impairing of anti-tumor immune responses	-	Lymphocyte-associated antigen 1 (LFA1) expression and CD8 T lymphocyte activity regulation	24

Upstream control of MAP4K4 in tumor cells is exerted by striatin (STRN) family proteins (members of the STRIPAK family). In an *in vitro* model of medulloblastoma, MAP4K4 interacted with STRN3/4 and stimulated cell growth. Accordingly, STRN3/4 depletion could reduce the invasive capacity of medulloblastoma cells (25). The interaction between MAP4K4 and STRN may be mediated by protein phosphatase 2A (PP2A). Recent experiments have demonstrated that STRN4 promotes MAP4K4 inactivation through the phosphatase activity of PP2A. Therefore, low levels of PP2A activity may contribute to cancer development (26). Other important upstream regulators of MAP4K4 function in tumor cells are TNFR, c-MET, PYK2, RAP2 among many others (27)

## MAP4K4 as a new therapeutic target in cancer

Evidence pertaining to the anti-neoplastic role of MAP4K4 inhibition is early but promising. Most studies in this area have been *in vitro* investigations leveraging RNA interference to block MAP4K4 function.

In a murine model of pancreatic cancer, specific pharmacological inhibition of MAP4K4 with GNE-495 inhibited pancreatic cell growth and tumor migration (17). MAP4K4 is also overexpressed in patients with pancreatic cancer and could represent a biomarker associated with poor clinical prognosis (17). In parallel, *in vitro* results demonstrated that inhibition of MAP4K4 (through the use of RNA interference with miR-98-5p) reduced the proliferation of pancreatic cancer cells (28).

In an *in vitro* model of glioblastoma multiforme, MAP4K4 inhibition reduced the invasion of tumor cells (23). MAP4K4 also reduced the chemosensitivity of cervical cancer cells to platinum therapy by regulating SOX6-induced autophagy (29). These results suggest that MAP4K4 inhibitors or specific autophagy inhibitors may increase the sensitivity of cervical cancer to chemotherapy. Additionally, the use of RNA interference (miR-200c) to block MAP4K4 in cervical cancer diminished the invasive behavior of the cancer cells *in vitro*. These results reinforce the idea that MAP4K4 inhibition may be a therapeutic target for cervical cancer treatment (30).

In colorectal cancer, MAP4K4 inhibition *via* miR-141 increased tumor cell chemosensitivity and diminished their proliferation, invasion, and migration (31). In breast cancer, miR-141 inhibited tumor cell proliferation by suppressing MAP4K4 expression, which was associated with an increase in tumor infiltration by CD4^+^ T lymphocytes (32). Thus, MAP4K4 inhibition could have anti-neoplastic effects in breast cancer by increasing immune cell infiltration. Furthermore, MAP4K4 inhibition provoked tumor cell apoptosis (by increasing the Bax/Bcl-2 ratio) and inhibited tumor cell proliferation in a gastric cancer model (15).

It is important to consider some caveats about the potential use of MAP4K4 inhibition as a therapeutic agent in cancer, because MAP4K4 has a role in multiple fundamental signaling systems, including NFκB activation, regulation of small GTPases and the Hippo cascade (33). In this line, a remarkable finding is the antiproliferative activity of MAP4K4 through activation of Hippo tumor suppressor signaling, which could lead to increased proliferation due to shutdown of Hippo signaling (34). Also, it is important to consider the potential adverse effects of a MAP4K4 inhibitor therapy. In this line, preclinical studies have reported that MAP4K inhibitors could be associated with weight loss, increased body temperature, tachycardia, among others (35). Thus, this new and promising area of research raises key questions that need to be addressed in the future before being implemented as a therapy.

**Figure 1:**
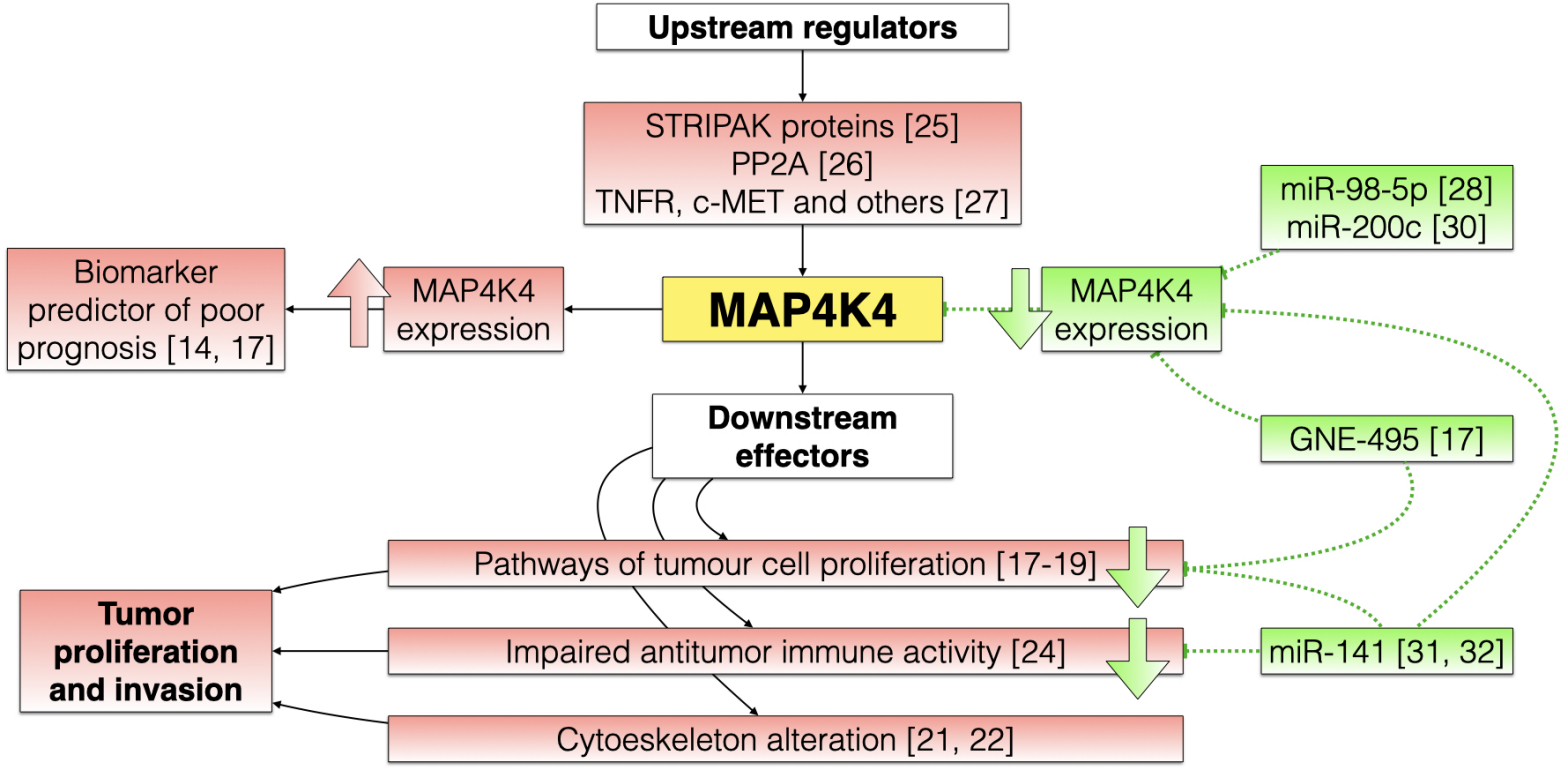
Schematic flow chart showing the possible pathways by which MAP4K4 can induce the initiation and progression of cancer, and the possible mechanisms by which MAP4K4 inhibitors could exert their antitumor effect. Oncogenic pathways are marked in red, and the mechanisms of MAP4K4 inhibitors are marked in green.

**Figure 1** shows a flow chart about the main ways by which MAP4K4 can stimulate tumor growth and invasion and the functions that specific MAP4K4 inhibitors could play.

## Discussion

Studies published over the last five years have shown that MAP4K4 plays a role in the initiation and progression of cancer, primarily by activating intracellular proliferative signaling (such as the JNK and MLK3 pathways), impairing cytoskeleton function, and reducing anti-tumor immune responses. However, the functions of MAP4K4 in cancer are just beginning to be described and could be more diverse than current data indicate. Multiple experiments with RNA interference reinforced the notion that this molecule is susceptible to pharmacological inhibition with specific inhibitors such as GNE-495. Is MAP4K4 ready for the main stage as a cancer therapy target? Although the current evidence is still early, it is promising. Further studies are needed to elucidate the efficacy of specific pharmacological MAP4K4 inhibition in cancer patients and to test the safety of its pharmacological blockade.

## Author contributions

All authors listed have made a substantial, direct, and intellectual contribution to the work and approved it for publication.
